# Ciprofloxacin and risk of hypolycemia in non-diabetic patients

**DOI:** 10.1186/s13256-019-2083-y

**Published:** 2019-05-12

**Authors:** Abiel Berhe, Mulugeta Russom, Fithawit Bahran, Goitom Hagos

**Affiliations:** 1 Department of Diagnostic Imaging, Orotta National Referral Hospital, Asmara, Eritrea; 2 Eritrean Pharmacovigilance Center, Asmara, Eritrea; 3 Department of Pharmacy Practice, Thumbay Hospital, Gulf Medical University, Ajman, United Arab Emirates; 4 Department of Internal Medicine, Orotta National Referral and Teaching Hospital, Asmara, Eritrea

**Keywords:** Ciprofloxacin, Hypoglycemia, Nondiabetic patients, VigiBase®

## Abstract

**Background:**

Fluoroquinolones have been associated with hypoglycemia in patients taking diabetic medications, most commonly due to drug-drug interactions and other associated risk factors. Except for four published case reports, there are no studies that have found positive associations between ciprofloxacin and hypoglycemia. In all but one of the cases, ciprofloxacin was taken with other hypoglycemic drugs. Recently, the Eritrean National Pharmacovigilance Centre received a serious case of hypoglycemia with recurrent episodes in a young and healthy patient without diabetes following use of oral ciprofloxacin. The aim of the present study is therefore to assess the causal relationship between ciprofloxacin and hypoglycemia in patients without diabetes using the World Health Organization-Uppsala Monitoring Centre global adverse drug reaction database (VigiBase®).

**Methods:**

A search was made on the World Health Organization global adverse drug reaction database (August 15, 2018) using “ciprofloxacin” as the drug substance and “hypoglycemia” as the reaction term. Cases that used hypoglycemic drugs (patients with diabetes) concurrently with ciprofloxacin and those with a completeness score below 50% were excluded to control for confounders and to improve the strength of the data. Hill criteria were used to assess causation.

**Results:**

A total of 35 cases of hypoglycemia reported since 1989 from 17 countries in patients without diabetes associated with ciprofloxacin use with a median time to onset of 4 days were retrieved. The cases have a median age of 64 years (interquartile range, 50–85) with a similar male–to-female ratio. Ciprofloxacin was the only suspect and the sole drug administered in 48.5% of the cases. In ten cases, hypoglycemia abated following withdrawal of ciprofloxacin, and reaction recurred in one case on the subsequent rechallenge. Hypoglycemia was marked as “serious” in 20 cases, and the outcome was fatal in two cases.

**Conclusions:**

This assessment found a suggestive causal link between use of ciprofloxacin and hypoglycemia in patients without diabetes.

## Background

Fluoroquinolones, the commonly used antibacterials, have been associated with dysglycemia (hypoglycemia and/or hyperglycemia) mainly in patients with diabetes taking either oral hypoglycemic agents or insulin [1]. Most of the patients who developed hypoglycemia following fluoroquinolone use had risk factors such as old age, diabetes, renal insufficiency, and concomitant use of hypoglycemic drugs, especially sulfonylureas [2–4].

An observational study reported an increased risk of dysglycemia with the use of fluoroquinolones in both patients with diabetes and patients without diabetes [5]. In that study, unlike with other fluoroquinolones, no cases of hypoglycemia were reported with the use of ciprofloxacin, though several cases of hyperglycemia were documented. A systematic review [6] and a retrospective cohort study [7] reported a higher risk of hypoglycemia associated with gatifloxacin but not with ciprofloxacin that resulted in gatifloxacin being pulled from the U.S. market [2]. Besides, in published case reports, levofloxacin has been associated with hypoglycemia, including death [8, 9]. In addition, on July 10, 2018, the U.S. Food and Drug Administration (FDA), in its review of postmarket pharmacovigilance data, communicated a safety alert on fluoroquinolones and risk of hypoglycemia [10].

The summary of product characteristics of ciprofloxacin registered with the FDA [9], European Medicines Agency [10], and Medicines and Healthcare Products Regulatory Activities [11] documents hypoglycemia as an adverse effect in patients with diabetes, possibly due to drug-drug interaction [11–13] or hyperinsulinemia [14]. In all cases, hypoglycemia abated shortly following withdrawal of ciprofloxacin. There was also a published case report of hypoglycemia following use of ciprofloxacin in an elderly patient without diabetes who had renal failure that immediately improved following withdrawal of the drug [15]. To the best of the authors’ knowledge, ciprofloxacin has not been associated with hypoglycemia in young, healthy patients without diabetes.

Recently, the Eritrean National Pharmacovigilance Centre received a serious case with recurrent episodes of hypoglycemia in a young, previously healthy patient without diabetes following use of oral ciprofloxacin. The aim of the present study therefore was to assess the causal relationship between ciprofloxacin and hypoglycemia in patients without diabetes using the World Health Organization (WHO) global adverse drug reaction database (VigiBase®).

## Methods

On August 15, 2018, a search was made in the WHO global suspected adverse drug reactions (ADR) database (VigiBase®), which is developed and maintained by the Uppsala Monitoring Center (UMC), Sweden. As of September 13, 2018, the database contained over 18 million individual case safety reports (ICSRs) submitted from 130-plus countries since 1968 [16].

The search criteria used were “ciprofloxacin” as the drug substance and “hypoglycemia” (high-level group term) as the reaction MedDRA (Medical Dictionary for Regulatory Activities) term. Reaction outcomes, seriousness, information component (IC), de-challenge and rechallenge, and other relevant information were retrieved using VigiLyze, UMC’s tool developed to analyze data from VigiBase. The IC value is a measure of disproportionality of drug-ADR in VigiBase as discussed by Bate *et al.* and Norén *et al*. [17, 18]. A positive IC value is a traditional threshold that indicates a drug-ADR pair is reported more often than expected based on all reports in VigiBase, thus showing a statistical signal. The results generated were then exported to an Excel 2016 spreadsheet (Microsoft, Redmond, WA, USA) for descriptive analysis. Potential duplicate cases were eliminated using VigiMatch™, a tool developed by the UMC.

Patients on antidiabetic medications and/or known to have a history of diabetes were excluded from the study to rule out the effect of hypoglycemic agents. Moreover, cases with completeness scores less than 50% were also excluded to improve the strength of information.

Hill’s criteria were used to assess causation, whereas “labeled-ness” or expectedness of the adverse event was checked by referring to the summary of product characteristics of ciprofloxacin and reviewing medical literature [19].

## Results

In this section, the complete description of the single case of hypoglycemia reported from Eritrea and the characteristics of similar reports retrieved from VigiBase are presented.

## Case presentation

A 28-year-old nonalcoholic and previously healthy businessman from the Tigrigna ethnic group, weighing 56 kg and with no previous exposure to ciprofloxacin, presented with hypoglycemia following intake of ciprofloxacin 500 mg oral twice daily for the treatment of urinary tract infection. He presented with chief complaints of blurred vision, headache, excessive sweating, and extreme hunger 4 days after the commencement of ciprofloxacin (December 4, 2017), which were relieved by consuming food. The next day, he went to a nearby clinic, and his 2-hour postprandial blood sugar level was 61 mg/dl with normal physical examination findings. Later the patient had recurrent episodes of hypoglycemia (four to five episodes per day). The event was manifested by diaphoresis drenching his body, blurry vision, and headache with blood sugar level ranging from 61 to 89 mg/dl measured postprandially at home.

After 48 hours, the patient became unconscious while at home and was admitted to an emergency department of the national referral hospital. On admission, the patient’s random blood sugar (RBS) was 55 mg/dl, and his physical findings were nonrevealing, although he was given juice and was administered 5% dextrose in water intravenously. Later, the patient was discharged from the hospital after he regained consciousness, and his RBS was measured as 89 mg/dl. During that time, the patient had not stopped taking ciprofloxacin, because it was not determined to be the possible cause of hypoglycemia.

In the 2 weeks following discharge from the emergency department, he experienced five to eight episodes of postprandial hypoglycemia per day (53– 88 mg/dl), which were relieved by intake of food. As a result, he was taking food every 2 hours to prevent hypoglycemia and was unable to leave his home for several days because of his fear of hypoglycemic episodes.

Following the second week, he visited a physician again because his symptoms were becoming debilitating. A complete history and physical examination, blood count, blood chemistry, ultrasound of the abdomen, and abdominal computed tomography (to rule out insulinoma) were performed. The results were found to be normal except for mild elevation of liver enzymes and lipid panel (blood urea nitrogen [BUN] = 4 mg/dl, creatinine = 1 mg/dl, aspartate aminotransferase [AST] = 116 unit/l, alanine aminotransferase [ALT] = 185 unit/l, alkaline phosphatase [ALP] = 82 unit/l, glucose = 69 mg/dl). In addition, the patient’s hemoglobin A1C level was found to be 5.3%. The patient’s insulin level was not determined because the test was not available. The patient was then admitted for further investigation and was closely followed with feeding every 2 hours and 6-hourly monitoring of his blood glucose level. Furthermore, the patient was taking 5% dextrose in water intravenously every 8 hours as maintenance. During his stay in the hospital, his RBS was 43 to 98 mg/dl. Later, the patient was discharged after 12 days of admission because his hypoglycemic episodes had improved to one to two times per day with a tentative diagnosis of hypoglycemia secondary to ciprofloxacin use. He was followed weekly for 1 month. One month later, his follow-up chemistry showed improvement in liver function, but his low blood glucose level persisted (BUN = 14 mg/dl, creatinine = 0.6 mg/dl, AST = 37 unit/l, ALT = 81 unit/l, ALP = 76 unit/l, albumin = 5.1 mg/dl, glucose = 31 mg/dl, uric acid = 5.7 mg/dl).

Six weeks after discharge, there were no episodes of hypoglycemia, and his RBS was normal for about 7 weeks. During the earlier distressing situation, his body weight had dramatically increased from 56 to 83 kg, which dropped to 58 kg 10 weeks after full recovery. The patient was then considered fully recovered from his recurrent hypoglycemia. According to the Naranjo probability scale [20], the event was found to be probably associated with ciprofloxacin.

### Reports in the WHO global database

**Fig. 1:**
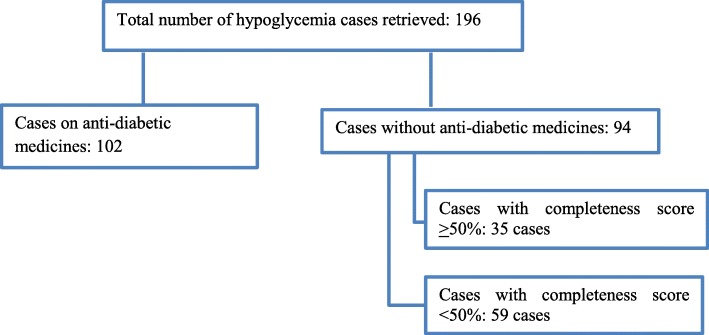
Inclusion criteria and number of cases retrieved from the global individual case safety reports database

To place our report in context, we conducted a review of previously reported cases in the global ICSR database (VigiBase). From 1989 to August 15, 2018, a total of 196 cases of hypoglycemia associated with ciprofloxacin, including 12 deaths, were submitted to the global ICSR database. The cases were reported from 17 countries across 5 continents. Excluding the cases with concurrent use of antidiabetes drugs and completeness scores below 0.5 or 50%, a total of 35 cases of hypoglycemia associated with ciprofloxacin were identified (Fig. 1). The male-to-female ratio was similar with a median age of 64 years (Table 1).

**Table 1:** Distribution of cases of hypoglycemia associated with ciprofloxacin, according to background characteristics

Background characteristics	No. (%)
Age at index date	
< 45 years	6 (17)
45–64 years	12 (34)
> 65 years	17 (49)
Median age (IQR)	64 (50–85)
Sex	
Male	18 (51.4)
Female	17 (48.6)

*IQR* Interquartile range

Ciprofloxacin was the only suspect and the sole drug administered in 48.5% (17 of 35) of the cases. In ten cases, hypoglycemia abated following withdrawal of ciprofloxacin, and the reaction recurred in one case on the subsequent rechallenge (readministration) of ciprofloxacin. Of the 35 cases, the median time to onset was 4 days following the commencement of the suspected drug, ciprofloxacin. Hypoglycemia was marked as “serious” in 20 cases, and the outcome of hypoglycemia was reported as fatal in two cases, recovered in 17, recovering in 2, not recovered in 2, and unknown in the rest of the cases.

The top coreported reactions were drug interaction, acute kidney injury, fatigue, hypotension, and confusional state in 5–13% of the cases. The commonly coreported drugs were lansoprazole and fosfomycin (three cases [8.3%] each) as well as cefuroxime, paracetamol, and pantoprazole (two cases each [5.5%]). The association has an IC value of − 0.62 and IC_025_ − 0.84. The results of the causality assessment of ciprofloxacin and hypoglycemia using Hill’s criteria are summarized in Table 2.

**Table 2:** Results of causality assessment on ciprofloxacin and hypoglycemia in patients without diabetes using Hill’s criteria

	Criterion	Outcome
1.	Strength of association	IC value was negative (no statistical signal).
2.	Consistency of the cases	Cases of hypoglycemia associated with ciprofloxacin in patients without diabetes have been reported to the WHO global database from different geographical areas. Moreover, there is a similar published case report of hypoglycemia in an elderly patient without diabetes following intake of ciprofloxacin. In many of the cases, hypoglycemia manifested shortly (within 4 days) following used of ciprofloxacin and recovered on de-challenge in a substantial number of cases.
3.	Specificity of the association	In 48.5% of the cases, patients took ciprofloxacin and shortly encountered hypoglycemic episodes without other coreported reactions.
4.	Temporal relationship	The time to reaction onset of hypoglycemia in a majority of the cases was within 4 days following intake of ciprofloxacin and when patients had no history of such events before. This shows that the association had a plausible temporal relationship.
5.	Dose-response relationship	No evidence of dose-response relationship was found on this association.
6.	Biological mechanism or plausibility	The possible mechanism that explains the causal association of ciprofloxacin and hypoglycemia could be increased insulin release via blockade of ATP-sensitive potassium channels in the beta-cells of the pancreas [21, 22].
7.	Experimental evidence	Two experimental studies in rat islet cells exposed to quinolones showed increase in insulin secretion via blockade of ATP-dependent potassium channels [21, 22]. Besides, the facts that hypoglycemia abated in ten cases following withdrawal of ciprofloxacin and that there was recurrence of the same event in one case after subsequent exposure to ciprofloxacin support the association.
8.	Analogy	Hypoglycemia is a well-known adverse effect of fluoroquinolones that have structural analogues with ciprofloxacin.
9.	Coherence	Taking into consideration the effect of fluoroquinolones in causing hyperinsulinemia in rat islet cells and the aforementioned preexisting knowledge, the association is coherent with today’s knowledge.

*Abbreviations: ATP* Adenosine triphosphate, *IC* Information component, WHO World Health Organization

## Discussion

Unlike other fluoroquinolones, the association of ciprofloxacin and hypoglycemia in patients without diabetes is not well documented, and studies conducted previously did not show a statistically significant association [6, 7]. This study, however, found a suggestive causal association between ciprofloxacin and hypoglycemia in patients without diabetes, which strengthens the FDA recommendation provided in July 2018 [10]. The facts that the majority of cases were reported from different geographical areas, the temporal relationship was plausible, and hypoglycemia was encountered in a substantial number of cases solely after administration of ciprofloxacin support a causal association. Besides, the positive de-challenge and rechallenge documented in some cases and the specificity of the association support the causation.

Two experimental studies in rat islet cells exposed to quinolones showed an increase in insulin secretion via blockade of adenosine triphosphate (ATP)–dependent potassium channels [21, 22]. Thus, the possible mechanism that explains the causal association of ciprofloxacin and hypoglycemia could be increased insulin release via blockade of ATP-sensitive potassium channels in the beta-cells of the pancreas. Other experimental studies using rat islet cells also suggest that fluoroquinolones act not as initiators but as augmenters of stimulated insulin release from these cells [23], which might be a reason that the majority of the episodes of hypoglycemia in our patient were occurring postprandially. Although ciprofloxacin is known to inhibit CYP1A4 isoenzymes that lead to drug interactions, especially with diabetic drugs such as glyburide [24], none of our cases were taking diabetic drugs, and ciprofloxacin was the sole drug used in about half of them.

The availability of the plausible biological mechanism, existence of experimental evidence, analogy, coherence, and reversibility of hypoglycemia in a majority of the cases following withdrawal of ciprofloxacin are also elements that support a causal relationship between ciprofloxacin and hypoglycemia.

Because the cases were voluntarily reported from different parts of the world, we cannot estimate the incidence rate of hypoglycemia associated with ciprofloxacin, which is the inherent limitation of spontaneous reporting of adverse drug reactions. Another limitation of this study is that insulin level was not determined, and because there was not any coreported diabetic drugs in the 35 cases, we assumed that the patients were not diabetic, which could also lead to exposure misclassification bias. Moreover, although a substantial number of the events were marked as “serious,” we do not know the degree, duration, and/or reversibility of hypoglycemia in some of the cases.

## Conclusions

This study suggests that ciprofloxacin can cause hypoglycemia even in nondiabetic and previously healthy patients. The seriousness of hypoglycemia and the few deaths reported also underscore the importance of early detection and appropriate management of patients with hypoglycemia. In cases of hyperinsulinemia, use of octreotide has been shown to be effective in abating recurrent hypoglycemic episodes associated with fluoroquinolones [14].

Taking the risks of hypoglycemia and other previously identified neuropsychiatric adverse effects [25] into consideration, clinicians should avoid the prescription of fluoroquinolones to patients who have other treatment options for acute bacterial sinusitis, acute bacterial exacerbation of chronic bronchitis, and uncomplicated urinary tract infections because the risks outweigh the benefits in these patients [10]. Further studies are therefore required to substantiate this safety signal and to identify possible risk factors that expose patients to hypoglycemia.

## Abbreviations

ADR: Adverse drug reaction
ALP: Alkaline phosphatase
ALT: Alanine aminotransferase
AST: Aspartate aminotransferase
ATP: Adenosine triphosphate
BUN: Blood urea nitrogen
CT: Computed tomography
FDA: U.S. Food and Drug Administration
IC: Information component
ICSR: Individual case safety report
IQR: Interquartile range
MedDRA: Medical Dictionary for Regulatory Activities
RBS: Random blood sugar
UMC: Uppsala Monitoring Center
WHO: World Health Organization
